# Establishment of Diagnostic Reference Levels in Cone Beam Computed Tomography Scans in the United Arab Emirates

**DOI:** 10.3390/tomography8060247

**Published:** 2022-12-14

**Authors:** Mohamed M. Abuzaid, Wiam Elshami, Deepa Jayachandran, Noushad Korappil, Huseyin O. Tekin

**Affiliations:** 1 Medical Diagnostic Imaging Department, College of Health Sciences, University of Sharjah, Sharjah 27272, United Arab Emirates; 2 Primary Health Care, Medical Support Services, Dubai Health Authority, Dubai 14660, United Arab Emirates; 3 Computer Engineering Department, Faculty of Engineering and Natural Sciences, Istinye University, Istanbul 34396, Turkey

**Keywords:** (CBCT) cone-beam computed tomography, (DRL) diagnostic reference level, effective dose, organ dose

## Abstract

This study aimed to address the knowledge gap in assessing the radiation doses from cone beam computed tomography (CBCT) procedures, establishing a typical value, and estimating effective and organ doses. A total of 340 patients aged 18–80 years were included in this study. Organ doses were estimated using VirtualDose IR software. The typical values were based on median values estimated as 1000 mGy cm^2^. The mean ED (µSv) per procedure was 149.5 ± 56, and the mean of the peak skin dose during the CBCT examination was 39.29 mGy. The highest organ dose was received by the salivary glands (2.71 mGy), the extrathoracic region (1.64 mGy), thyroid (1.24 mGy) and eyes (0.61 mGy). The patients’ doses were higher than in previous studies. Staff awareness, education, training and dose optimisation are highly recommended. With the establishment of local DRLs, patient dosages can be reduced successfully without compromising image quality.

## 1. Introduction

Radiology examinations expose patients to a significant dose of ionising radiation, which carries both radiogenic risks and the advantage of a correct diagnosis for various clinical diseases. Radiological examinations have been identified as a significant source of patient exposure that raises the overall dosage received by the general public. Globally, it was calculated that the collective effective dose (ED) was 4.2 million man-Sv. Dental radiology procedures are one of the highest-frequency procedures and represent 26.3% (≈1.0 billion) of the total radiological procedures and 0.2% (8.4 million man-Sv) of the total collective dose per procedure [1]. Therefore, the increasing number of radiographic procedures should be carefully investigated. 

Ionising radiation’s stochastic effects are chance occurrences, with the likelihood of an effect growing with dose, but the impact of an effect is unrelated to dose. The emergence of cancer in an irradiated organ or tissue is a very unusual stochastic consequence. The dose received is often inversely correlated with the chance of occurrence. Many years after radiation exposure, stochastic effects are evident (the latent period) [2]. 

Cone-beam computed tomography (CBCT), a 3D technology, has revolutionised the profession of oral and maxillofacial radiology since its introduction in clinical applications in 1996 [3]. Compared to computed tomography (CT), CBCT was quickly adopted in dentistry due to the small size of equipment, low cost, fast acquisition time, wide coverage area and low number of motion artefacts. In addition, CBCT provides a 3D examination of the craniofacial area with minimum distortion. The diagnostic reference level (DRL) establishment is recommended by the International Commission on Radiological Protection (ICRP) for each country and imaging procedure and should be reviewed regularly. Diagnostic reference levels are frequently used to express measurable doses from radiological procedures in the context of dose optimisation. They provide an indication of the radiation dose that may be received by a patient undergoing a particular imaging procedure. Imaging facilities can identify procedures that might be amenable to further optimisation by comparing typical (median) exposure levels for popular imaging procedures. Based on the typical values or DRL, action can be taken to optimise the dose [4,5].

It is important to ensure that patient exposure is as low as reasonably achievable. In the CBCT procedure, the development of specific imaging protocols customised to the patient’s age or weight, organ of interest and clinical indication must be used [6]. Radiation dose estimations from several CBCT machines have revealed significant dose differences between different departments for the same examination and identical patient groups. These findings indicate the necessity of establishing a DRL and decreasing dose variance without sacrificing clinical outcomes [7,8]. Setting DRLs in diagnostic radiology is just the beginning of a long process; DRLs help to identify facilities where optimisation of protection is required. A local review of practices and equipment should be conducted to determine whether the protection has been optimised. Steps should be taken to address any deficiencies if the median dose from a hospital survey exceeds the DRL. This calls for knowledgeable and skilled personnel to advise on and implement the adjustments [4]. 

The establishment of DRLs in the United Arab Emirates (UAE) is progressing in different imaging modalities, with the support of a national radiation protection organisation [9,10]. However, UAE national DRLs are not yet available for dental CBCT procedures. This study addressed a knowledge gap in assessing the radiation doses from CBCT procedures, establishing typical values and estimating effective and organ doses. This is a pilot study and the first effort to establish DRL for CBCT; the results of this study will be used as a guide for future efforts and suggested values will be recommended for dose optimisation. 

## 2. Materials and Methods

### 2.1. Patient Population 

The study was conducted at primary healthcare centres (PHCs) in Dubai Healthcare Authority (DHA); the PHCs comprised 13 centres throughout the Emirate of Dubai, offering a ratio of one health centre for every 30,000 individuals. Retrospective data were collected from patients undergoing CBCT after procedure justification by dental specialists. This study evaluated patients for endodontics, root resorption and pathological conditions. The indications included localisation of the mandibular canal, luxated teeth and dental implants, assessing the anatomical relationship in root canal filling materials, lesion assessment and mandibular or condylar fractures. Previous research and recommendations sample size recommended 10 and 20 patients for each protocol [8]. The study sample consisted of 340 adult patients undergoing CBCT performed by dental radiographers for four months (April–August 2021). 

### 2.2. CBCT Unit

One Planmeca ProMax 3D Max CBCT machine was used for all dental exams (Planmeca OY, Helsinki, Finland). Regular quality measurement and quality control activities were implemented. The Planmeca ProMax 3D Max offers an ultra-low dosage imaging technique with pulsed exposure capabilities (10–20 ms) to provide dose optimisation as low as reasonably attainable (ALARA) without affecting CBCT picture quality. The total filtration (Al) consisted of 2.5 + 0.5 Cu extra filtration and a focus point of 0.5 mm on the tube. Table 1 lists the machine’s characteristics.

### 2.3. Radiation Dosimetry 

The ICRP recommends using PKA, Ka,r, CTDIvol and dose-area product (DAP) as DRL quantities, depending on availability [8]. The machine measured in DAP; therefore, values are reported as such. For the DRL’s calculation, we used the dose-area product (DAP, Gy·cm^2^). In this calculation, we aimed to establish the typical values according to what represented a typical local practice at a single large centre or group of healthcare facilities, and the typical value set was the median value of the distribution of doses determined from patient samples [8]. 

### 2.4. Organ Dose 

The web-based VirtualDose CT software tool (Virtual Phantoms, Inc., New York, NY, USA) was used to estimate the effective and organ dose https://www.virtualphantoms.com/, accessed on 8 February 2022. The software was developed at the Rensselaer Polytechnic Institute and the University of Florida using computational phantoms [11,12]. The organ dose was calculated for each procedure. The organ dose calculation was based on two phantoms: an adult male and a female. The parameters used for the calculations were scan protocol, CT manufacturer, scanner name, kVp and mAs [13]. 

## 3. Results

Three hundred and forty adult patients were enrolled in the study, comprising 174 (52.2%) female and 166 (48.8%) male patients. The patients’ ages ranged from 18 to 80 years, with a mean and standard deviation (SD) of 39.4 ± 18.5 years. The exposure parameters used during the procedures were a fixed tube voltage (90 kVp), a current tube range between 8–14 mA and exposure time ranging between 12 and 16 s (Table 1). 

**Figure 1:**
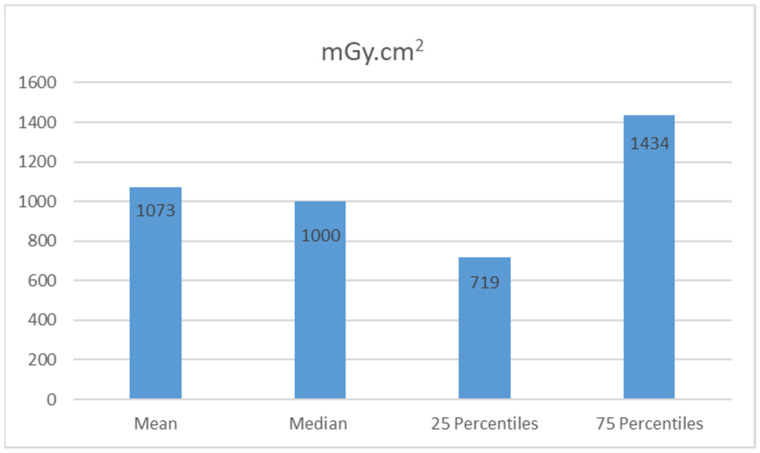
Patients’ dose (mode, median, range and percentile).

Figure 1 shows the descriptive statistics of the patients’ doses. The DAP values were: mean 1073 mGy·cm^2^, median 1000 mGy·cm^2^, (50th percentile) and 75th percentile 1434 mGy·cm^2^. The proposed typical value in this study was based on dose distributions obtained from a local dose survey, based on the median value of a single patient dose per examination and estimated to be 1000 mGy/cm^2^.

**Figure 2:**
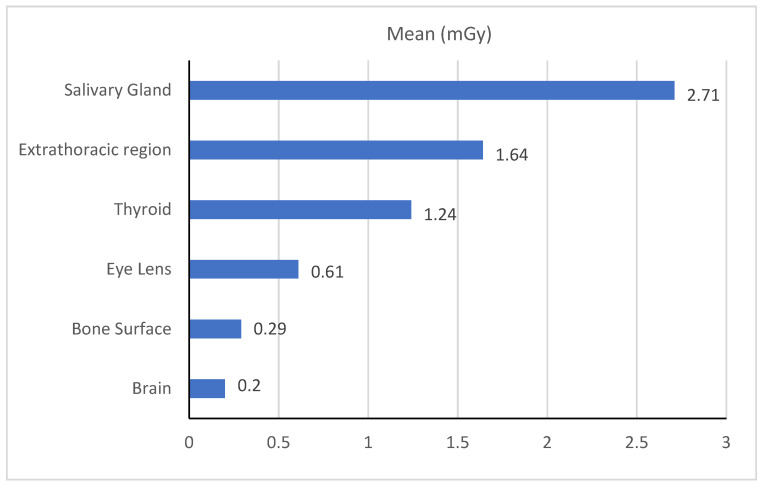
Mean organ dose (mGy) during CBCT procedure.

The current study calculated the effective dose based on tissue weighting factors and the organ equivalent dose using DAP [14]. The mean effective dose per procedure was 149.5 ± 56 µSv. In this study, the organ doses to the brain, bone surface, eyes lens, thyroid, extra thoracic region and salivary glands were 0.2, 0.29, 0.61, 1.24, 1.64 and 2.71 mGy per procedure, respectively (see Figure 2). 

## 4. Discussion

There are no standard exposure parameters for CBCT, as vendors use different detectors and exposure settings [11,12]. Therefore, it is recommended that the radiation dose be optimised to ensure that patients receive the minimum achievable dose for the diagnostic task. The exposure parameters in this study produced a range of patient doses between 573 and 1689 mGy·cm^2^ with up to a three-fold difference. The radiation range was low compared to previous studies, which produced ranges up to 10-fold and 20-fold higher, with kVp ranges of 71–120 [7,13]. The introduction of DRLs for CBCT in practice will facilitate the identification of high or low doses and consider the action of dose optimisation concerning image quality [10]. Various studies have reported effective dose variations in different CBCT machines, using exposure parameters, image receptors, filtration and image resolution factors. For example, in dental imaging, the effective dose from panoramic radiography ranges from 48.0 to 652.0 µSv, which is lower than the patient doses resulting from the CBCT procedure (68–1073 µSv) [15].

This study’s proposed typical values are comparable to those of various studies conducted between 2011 and 2020. It is clear that the median dose in this study, 1000 mGy·cm^2^, is higher than the doses in Japan (2013), Finland (2016), the UK (2017) and Switzerland (2020), which were 414, 380, 265 and 683 mGy·cm^2^, respectively. A recent study by the International Atomic Energy Agency (IAEA), conducted in nine East European countries, showed a result of 1000 mGy·cm^2^, which is similar to our results [16,17,18,19,20,21,22], Table 2.

Studies have shown that the use of DRLs has proven to be a useful quality assurance tool in medical imaging [23]. In the current study, the mean effective dose per procedure was 149.5 ± 56 µSv, which is equivalent to a study conducted in Saudi Arabia using a similar machine (142.8 ± 35 µSv) [24]. Ludlow et al. (2008) and Pauwels et al. (2012) estimated the organ dose during CBCT using TLD and an anthropomorphic phantom as 68.0–1073.0 and 28.0–560.0 µSv for the maxillofacial region and dentoalveolar region, respectively [16,23]. Qiang et al. reported that the effective dose of dental CBCT was equivalent to 200 µSv [24]. Higher organ doses of 630, 7700, 8700 and 4000 μGy were reported for the brain, salivary glands, thyroid gland and eye lens, respectively [16]. The values reported in this study are lower than those reported in previously published studies. Pauwels et al. recommended that CBCT should only be justified if it provides new information that cannot be obtained by panoramic radiography [23]. In addition, shielding, proper collimation and an optimised tube current-time product will reduce the dose absorbed in the thyroid and other organs. 

Although DRLs have been recognised as a valuable tool for dose reduction of up to 50%, limited studies are available regarding CBCT DRL. Furthermore, the current study also revealed a wide variation in international practices in CBCT DRL, up to four-fold from one country to another, suggesting that extensive effort is required to harmonise the imaging protocols to maximise diagnostic findings while reducing the number of patient doses [25].

The dose reduction technique is based on many factors, such as the justification of every individual case. When the examinations are justified, the dose saving arises from the optimisation of examinations. The dose optimisation principle is applied to an individual case to produce high image quality sufficient to provide a diagnosis with a low radiation dose. DRL’s role in practice is to reduce dose levels without degrading image quality or patient care [4].

## 5. Limitation

The study’s findings were based on information provided by a single machine (the one that was readily available). Additionally, this work relied on the accuracy of reported DAP values from the scanner, which is regularly checked for accuracy by both the manufacturer and the quality assurance department. 

## 6. Conclusions

The use of CBCT has increased in recent years, in line with the increasing exposure of the population to radiological procedures. However, until now, no national values have been available in the field of CBCT in the UAE [9,10]. This is the first survey of doses in CBCT and has given us a good initial view of how CBCT is being used in dental practices in the UAE and will be helpful in future optimisation. This study’s typical values for CBCT were higher than those in prior studies. The results will guide professionals involved in CBCT to enable safe practices.

On the other side, the findings will establish practice standards and help to better understand the radiation safety implications of CBCT. For instance, accurate documenting of each patient’s procedure data is crucial for calculating patient doses, particularly if a variation from the established protocol occurs. 

## Figures and Tables

**Table 1:** Planmeca ProMax 3D Max features and exposure parameters.

Parameter	Characteristics
Resolution	Normal
Isotropic voxel size (mm)	0.16–0.32
Tube voltage, mA	90 (kVp), 16 mA
Tube current-time product	158 ± 2.6 (16.0–196.0) (mAs)
Filtration	2.8 (mm Al)
Degree of rotation	3600
Dimension of collimator area	8 × 13 (cm^2^)
Dose optimisation	Ultra-Low Dose™ imaging protocol, Pulsed X-ray radiography

**Table 2:** Current study’s proposed dose compared to those of previous studies.

	Dose (mGy.cm^2^)
Current Study	1000
IAEA	1000
Japan (2013)	414
Finland (2016)	380
UK (2017)	265
Switzerland (2020)	638

## Data Availability

Not applicable.
